# Computational identification and characterization of chitinase 1 and chitinase 2 from neotropical isolates of *Beauveria bassiana*

**DOI:** 10.3389/fbinf.2024.1434442

**Published:** 2024-10-18

**Authors:** Juan Segura-Vega, Allan González-Herrera, Ramón Molina-Bravo, Stefany Solano-González

**Affiliations:** ^1^ Laboratorio de Bioinformática Aplicada, Escuela de Ciencias Biológicas, Universidad Nacional de Costa Rica, Heredia, Costa Rica; ^2^ Laboratorio de Control Biológico, Escuela de Ciencias Agrarias, Universidad Nacional de Costa Rica, Heredia, Costa Rica; ^3^ Programa de Biotecnología Vegetal y Recursos Genéticos para el Fitomejoramiento (BIOVERFI), Escuela de Ciencias Agrarias, Universidad Nacional de Costa Rica, Heredia, Costa Rica

**Keywords:** entomopathogen, bioinformatics, gene structure, fungi, physicochemical parameters, genomics, biocontrol

## Abstract

**Background:**

The fungus *Beauveria bassiana* is widely used for agronomical applications, mainly in biological control. *B. bassiana* uses chitinase enzymes to degrade chitin, a major chemical component found in insect exoskeletons and fungal cell walls. However, until recently, genomic information on neotropical isolates, as well as their metabolic and biotechnological potential, has been limited.

**Methods:**

Eight complete *B. bassiana* genomes of Neotropical origin and three references were studied to identify chitinase genes and its corresponding proteins, which were curated and characterized using manual curation and computational tools. We conducted a computational study to highlight functional differences and similarities for chitinase proteins in these Neotropical isolates.

**Results:**

Eleven chitinase 1 genes were identified, categorized as chitinase 1.1 and chitinase 1.2. Five chitinase 2 genes were identified but presented a higher sequence conservation across all sequences. Interestingly, physicochemical parameters were more similar between chitinase 1.1 and chitinase 2 than between chitinase 1.1 and 1.2.

**Conclusion:**

Chitinases 1 and 2 demonstrated variations, especially within chitinase 1, which presented a potential paralog. These differences were observed in their physical parameters. Additionally, CHIT2 completely lacks a signal peptide. This implies that CHIT1 might be associated with infection processes, while CHIT2 could be involved in morphogenesis and cellular growth. Therefore, our work highlights the importance of computational studies on local isolates, providing valuable resources for further experimental validation. Intrinsic changes within local species can significantly impact our understanding of complex pathogen-host interactions and offer practical applications, such as biological control.

## 1 Introduction

*Beauveria bassiana* (Ascomycota: Hypocreales) is a cosmopolitan fungus with the ability to survive as a saprophyte in soil and an as endophyte in plants; but can also act as an insect pathogen (Valero-Jiménez et al., 2016). The potential of this fungus to infect insects has been utilized in the agricultural industry to produce biopesticides (de Faria and Wraight, 2007). Unlike entomopathogenic viruses and bacteria, fungi do not infect insect hosts by ingestion. Instead, fungi initiate infection through penetration of the insect’s cuticle (Mascarin and Jaronski, 2016). To overcome the host’s cuticular layer, *B. bassiana* produces several enzymes capable of degrading cuticle constituents, among which proteases and chitinases stand out (Valero-Jiménez et al., 2016).

The conditions of the insect cuticle are considered adverse due to exposure to solar radiation, osmotic stress, and the insect’s defense responses. Therefore, rapid degradation of the cuticle would result in less exposure time to these conditions and thus a higher probability of infection success (Ortiz-Urquiza and Keyhani, 2016; Valero-Jiménez et al., 2016). For this reason, hydrolytic enzymes are considered important elements of pathogenesis. Chitinase is the most important enzyme in degrading the chitin polymer in the cuticular layer, and its activity has been associated with the virulence of entomopathogenic fungi (Pelizza et al., 2012; Dhawan and Joshi, 2017).

Chitinases are hydrolytic enzymes that degrade chitin into its oligo and monomeric components by hydrolyzing the β-1,4 N-acetyl-D-glucosaminide bonds (Le and Yang, 2019). According to their cleavage pattern, chitinases are divided into endo and exochitinases. Endochitinases can hydrolyze chitin at any point, generating products of variable size, while exochitinases do so from the non-reducing end of the chain, producing N,N′-diacetylchitobiose (Seidl, 2008). Additionally, based on the amino acid sequence, chitinases can be classified into families GH 18 and 19. These families show no sequence similarity to each other and differ in their catalytic mechanisms (Seidl, 2008).

Fungal chitinases not only play roles in pathogenesis, but also serve functions in morphogenesis, cell division, autolysis, among others (Le and Yang, 2019). The implication of chitinases in pathogenesis has prompted research development to enhance the understanding of these enzymes during the infection process. Despite numerous studies quantifying enzymatic production and gene expression encoding chitinases in *B. bassiana* (Fang et al., 2005; Pelizza et al., 2012; Al Khoury et al., 2019; Bhadani et al., 2021), there is still limited information regarding their structural and functional characteristics (Bhagwat et al., 2021). Several genomic resources are available for *B. bassiana* including whole genome sequences (Kim et al., 2016). More recently, eight assembled genomes for isolates of Neotropical origin have been published (Castro-Vásquez et al., 2022; Solano-González et al., 2023) and can serve as useful tools for comparison, as numerous organisms from this region are diverse.

The objective of this work was to compare, at an *in silico* level, gene sequences of two chitinases from Neotropical isolates of *B. bassiana* to determine their structural and functional characteristics to facilitate understanding of their biological functions. Furthermore, as building knowledge relying on previous genomic assemblies from these species (Solano-González et al., 2023), this study provides resources to further manipulate genetic sequences of chitinase through future experimental validation as a bottom line aiming to evaluate the potential of these fungi as biological control agents.

## 2 Materials and methods

### 2.1 Data obtention and chitinase coding sequences identification

Genomic sequences from six isolates of *B. bassiana* (Castro-Vásquez et al., 2022) belonging to the entomopathogenic fungi collection of the School of Agrarian Sciences (ECA) of the National University of Costa Rica (UNA) were used. Additionally, sequences from strains reported in the NCBI database were included as references (Supplementary Table 1).

**FIGURE 1:**
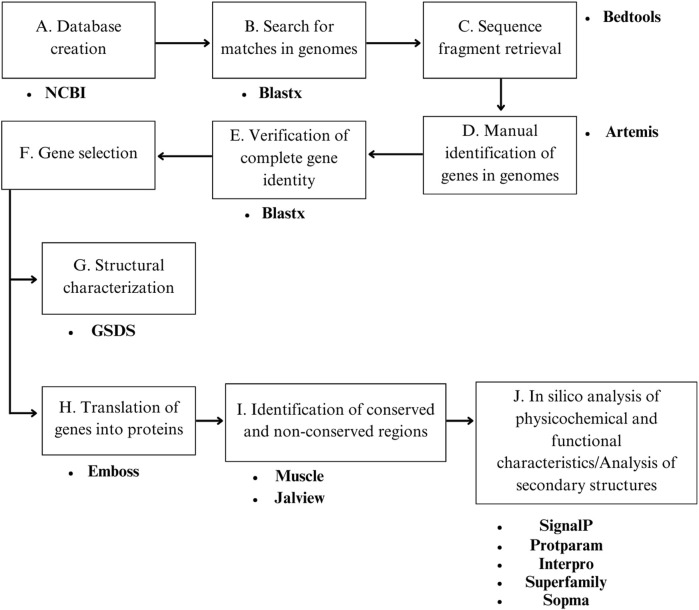
Schematic view of the methodology used to obtain the target sequences and their respective analyses. **(A)** Retrieval of chitinase protein sequences CHIT1 and CHIT2 from NCBI for database construction. **(B)** Identification of matches in the genomes of *B. bassiana* using Blastx. **(C)** Retrieval of sequence fragments using Bedtools v.2.27.1. **(D)** Manual identification of genes in the genomes using the Artemis tool v.18.0.0. **(E)** Verification of the identity of the complete gene sequences using an online Blastx. **(F)** Selection of genes based on coverage and identity criteria, annotation, and size. **(G)** Structural characterization using GSDS v2.0. **(H)** Translation of genes into proteins using the EMBOSS Transeq tool v.6.6.0. **(I)** Identification of conserved and non-conserved regions using Muscle v.3.8.31, Jalview v.2.11.6, and MEGA v.11. **(J)** *In silico* analysis of physicochemical and functional characteristics using SignalP, ProtParam InterPro, respectively; and analysis of secondary structures using Superfamily and SOPMA.

For the identification of chitinase coding sequences, a local blastx was performed on the *Laboratorio de Bioinformática Aplicada* (LABAP) computational platform at UNA. All CHIT1 and CHIT2 protein sequences of *B. bassiana* reported in the Identical Protein Groups resource of NCBI (https://www.ncbi.nlm.nih.gov/ipg) were used as the database (Figure 1B). Matches were filtered based on identity percentage (≥90%). Subsequently, sequence fragments were retrieved using the Bedtools v.2.27.1 tool (Quinlan and Hall, 2010) and manually mapped within the genomes using Artemis v.18.0.0 (Rutherford et al., 2000) (Figures 1C, D) (Supplementary Table 2).

To verify the identity of the complete gene and its correspondence with *chit1* and *chit2* genes, an online blastx was performed (https://blast.ncbi.nlm.nih.gov/Blast.cgi) using default parameters (Figure 1E). Only the top three hits were considered as they exhibited the highest identity and coverage percentages (Supplementary Table 2). Subsequently, a final selection of these genes was carried out, to exclusively include genes relevant to the objectives of the study (those most similar to *chit1* and *chit2*). The final selection was based on three criteria: 1) Genes whose identity and coverage percentage were ≥90 ± 3% with *chit1* or *chit2*, 2) Genes already annotated as chitinase 1 or chitinase 2, however, those annotated differently from *chit1* or *chit2*, were excluded, irrespective of whether they met the first criterion 3) Genes with a size greater than 3,000 bp were also discarded regardless of whether they satisfied the previous criteria (Figure 1F).

### 2.2 Chitinase structural *in silico* characterization

The structural annotation of the nucleotide sequences associated with *chit1* and *chit2* from the Neotropical fungal isolates was performed using GSDS v2.0 (http://gsds.gao-lab.org/). For this purpose, the complete sequences of each gene and their respective coding regions were used to visualize the location and size of introns and exons (Figure 1G).

### 2.3 Identification of conserved and non-conserved regions in *chit1* and *chit2*

Translation of the peptide sequences corresponding to *chit1* and *chit2* genes from the studied genomes was carried out using the EMBOSS Transeq tool v.6.6.0 (https://www.ebi.ac.uk/Tools/st/emboss_transeq/) using default settings (Figure 1H). These sequences were used in conjunction with reference sequences to construct a multiple-sequence alignment using Muscle v.3.8.31. With the aim of visualizing sequence similarity, a multiple alignment of peptide sequences was performed. The alignments included sequences from Neotropical isolates and sequences from reference strains, totaling 27 sequences of CHIT1 and 11 sequences of CHIT2, respectively. The visualization was performed in Jalview v.2.11.6 to determine conserved and non-conserved regions among the different sequences (Figure 1I). A distance matrix was constructed using the aligned sequences of *CHIT1* by replacing each amino acid with its numeric value based on relative entropy (Li et al., 2008). A hierarchical cluster dendrogram was drawn using the *hclust* function in RStudio (R Core Team, 2022).

On the other hand, for *CHIT2* a Maximum likelihood tree was constructed from *chit2* from *B. bassiana* draft isolates and references. This tree was constructed using Muscle alignments of 11 chit2 genes, implementing the JTT model and 1,000 replicates for bootstraps support, depicted in numbers. The tree shows Costa Rican isolates (BV-ECA 0, BV-ECA 26, and BV-ECA 31), Honduran isolate (BV-ECA 13), and the Puerto Rican isolate (BV-ECA 43) in addition to NCBI references.

### 2.4 *In silico* analysis of physicochemical and functional characteristics

SignalP6.0 (https://services.healthtech.dtu.dk/services/SignalP-6.0/) was used to predict the presence of potential signal peptides in all chitinases.

ExPASy ProtParam tool (https://web.expasy.org/protparam/) was used for the calculation and analysis of the physicochemical properties of the chitinases such as molecular weight, isoelectric point (pI), instability index (II), aliphatic index and the grand average of hydropathicity (GRAVY) were the evaluated.

The functional classification of the sequences corresponding to the Neotropical isolates was performed through a manual search and review of matches in protein databases associated with InterPro, for which its online version (https://www.ebi.ac.uk/interpro/search/sequence/) was used, implementing the default configuration (all selected databases). The SUPERFAMILY database (Pandurangan et al., 2019) was also used to determine the superfamily and subgroup associated with each of the chitinases (Figure 1J).

In addition, the prediction of secondary structures of chitinases was also carried out using the SOPMA web server (http://npsa-pbil.ibcp.fr/) (Geourjon and Deleage, 1995) maintaining the parameters established by default. Four conformational states were determined: helix, sheet, turn and coil.

A complete schematic view of the methodology is depicted in Figure 1.

## 3 Results

### 3.1 Data obtention and chitinase coding sequences identification

During data retrieval, a total of 50 genes similar to *chit1* and 11 genes similar to *chit2* were recovered (Supplementary Table 2). After the final selection process, 27 genes similar to *chit1* met the established selection criteria, set as: identity and coverage percentage of ≥90 ± 3%, absence of gene annotations different from *chit1* or *chit2*, as appropriate, and a size <3,000 bp (see Supplementary Tables 1, 2). Of these 27 genes, 11 belonged to the Neotropical isolates. As for the 11 *chit2* genes, all met the selection criteria; and 5 belonged to the neotropical isolates. Selected gene sequences, as well as their respective protein sequences, were used in subsequent analyses.

### 3.2 Chitinase structural characterization

The structural characterization of the sequences corresponding to the ECA-UNA isolates shows that gene composition is very similar in terms of the quantity and distribution of introns. Regarding genes similar to *chit1*, only gene g5795 from the BV-ECA 27 isolate, did not present intronic regions. All other genes contained two introns of 44 and 48 bp or 53 and 59 bp, except for gene g1800 from BV-ECA 31, which had introns of 45 and 75 bp. Genes g5795 and g1800 showed the greatest difference in length, at 867 and 1,185 bp, respectively, while all others had lengths of 1,274 or 1,462 bp (Supplementary Figure 1). On the other hand, none of the sequences similar to *chit2* from the neotropical isolates contain intronic regions. Furthermore, all these sequences present exactly a 1,044 bp size (Supplementary Figure 2).

### 3.3 Identification of conserved and non-conserved regions in CHIT1 and CHIT2

**FIGURE 2:**
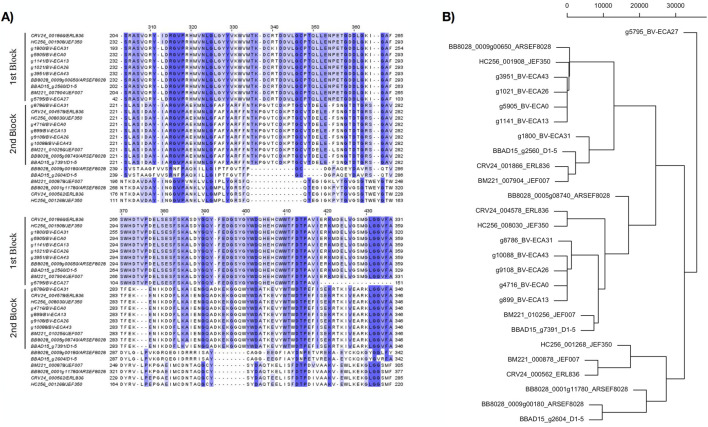
Sequence alignment and phylogeny of CHIT1. **(A)** Partial alignment region of peptide sequences like *chit1*. Colored sections represent residues that are similar or identical between sequences. A higher intensity in color indicates a greater conservation of residues among the sequences. Areas with dashed lines represent gaps. On the left side of the image is the name of the sequence, consisting of the gene ID followed by the isolate or strain it comes from. In the figure, two groups of sequences are highlighted by labels, which show similarity in most of the residues. The first block is denoted as chit1.1 and the second block are denoted as chit1.2. **(B)** Hierarchical cluster dendrogram constructed based on a distance matrix from the aligned sequences of CHIT1 by replacing each amino acid with its numeric value based on relative entropy.

Most sequences similar to CHIT1 are grouped into two large clusters (Figures 2A, B). The first group, hereinafter referred to as CHIT1.1, encompassed 10 sequences, five of which were from ECA fungal accessions (g3951_BV-ECA43, g1021_BV-ECA26, g5905_BV-ECA, g1141_BV-ECA13, and g1800_BV-ECA31), while the remaining accessions were from reference strains. The second group, named CHIT1.2, was composed of ten sequences, five of these originated from the Neotropical isolates (g8786_BV-ECA31, g10088_BV-ECA43, g9108_BV-ECA26, g4716_BV-ECA0, and g899_BVECA13). Sequence alignment for the first group and the second group exhibited high conservation of residues throughout the entire peptide chain. On the other hand, the six remaining sequences that were not grouped show lesser similarity to the sequences of both groups (Figures 2A, B). Hence, these peptide sequences may also correspond to different chit genes. However, none of the six remaining sequences correspond to Neotropical isolates. Additionally, the clustering supported by the dendogram show CHIT1.1 and CHIT1.2 groups (Figure 2B).

**FIGURE 3:**
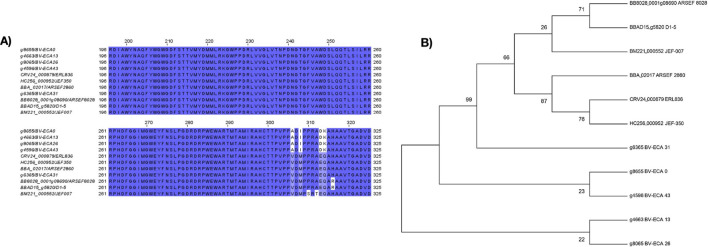
Sequence alignment and phylogeny of CHIT2. **(A)** Partial alignment region of peptide sequences similar to *chit2*. The colored parts represent regions that are similar or identical between sequences. A higher intensity in color indicates a greater conservation of residues among the sequences. On the left side of the image is the name of the sequence, consisting of the gene ID followed by the isolate or strain. **(B)** Maximum likelihood tree constructed from *chit2* genes from *B. bassiana* draft isolates and references. This tree was constructed using Muscle alignments of 11 chit2 genes, implementing the JTT model and 1,000 replicates for bootstraps support, depicted in numbers. The tree shows Costa Rican isolates (BV-ECA 0, BV-ECA 26, and BV-ECA 31), Honduran isolate (BV-ECA 13), and the Puerto Rican isolate (BV-ECA 43) in addition to NCBI references.

On the other hand, all CHIT2 sequences were highly conserved, with one or two amino acid substitutions. Therefore, both the peptide sequences from reference strains and the sequences from Neotropical isolates correspond to the same *chit2* gene (Figures 3A, B).

### 3.4 *In silico* analysis of physicochemical and functional characteristics

The presence of SEC/SPI signal peptides was predicted with a probability greater than 99% in the sequences of the CHIT1.1 group, except for g5795 BV-ECA 27 and g1800 BV-ECA 31. Additionally, the peptidase cleavage site of these chitinases was predicted between residues 21 and 22. In contrast with the CHIT1.2 group, the presence of signal peptides was predicted with a probability greater than 99% in all analyzed sequences, with cleavage site positions between residues 17 and 18. No signal peptides were detected in any of the sequences of the CHIT2 group.

The InterPro database was used to determine the classification and associated functions of the studied chitinases. All chitinases in the CHIT1.1 and CHIT1.2 clustered groups were classified as members of the glycosyl hydrolase family 18, except for chitinase g5795 from isolate BV-ECA 27, which was not assigned to any specific family. Additionally, members of the CHIT2 group were also not assigned to a particular family. However, the catalytic domain of glycosyl hydrolases was found in all three groups of chitinases.

In terms of functions, chitinases in the CHIT1.1 and CHIT1.2 groups were associated with the same GO terms: chitin binding and chitinase activity, except for g5795 BV-ECA 27, which again was not associated with any specific functional term. On the other hand, using the SUPERFAMILY database, all analyzed chitinases were grouped into the transglucosylases superfamily and classified as members of the type II chitinase group. Secondary structures of the chitinases were analyzed using the SOPMA tool (Supplementary Table 4). When comparing the ECA-UNA chitinases with the reference chitinases of each group, no significant variations were observed in the proportion of secondary structures, which coincided with the low standard deviation values. CHIT1.1 chitinases consistently displayed comparable proportions of alpha helix and random coil elements (37.82% and 39.76%, respectively), while beta sheets and beta turns were found in a smaller proportion (15.59% and 6.83%). On the other hand, CHIT1.2 and CHIT2 chitinases showed a greater difference between the proportion of random coil and alpha helix structures. In CHIT1.2, 46.88% of random coils and 34.62% of alpha helices were predicted, while in CHIT2, 44.81% of random coils and 30.23% of alpha helices were observed. Beta sheets were present in 13.02% in CHIT1.2% and 18.32% in CHIT2, while the proportion of beta turns was 5.49% and 6.63% in CHIT1.2 and CHIT2, respectively.

Regarding ExPASy ProtParam predictions, these showed chitinases from isolates BV-ECA 0, BV-ECA 13, BV-ECA 26 had the highest molecular weight (44000.79 Da) for the CHIT1.1 group, whereas the lowest weight was r found in isolate BV-ECA 27 (17344.35 Da), which did not group to either CHIT1.1 or CHIT1.2 groups. On the other hand, the molecular weight for chitinases from the CHIT1.2 group was very similar among all isolates, ranging from 49163.24 to 49198.42 Da. As for chitinase CHIT2, the estimated molecular weight for all sequences was 38501.57–38533.59 Da (Supplementary Table 3).

The isoelectric point (pI) was below 7 in all studied chitinases. For CHIT1.1, the pI value ranged from 4.72 to 4.94 among different isolates. For CHIT1.2, values between 5.16 and 5.42 were calculated, while the pI for chitinase CHIT2 was 4.64–4.68 (Supplementary Table 3).

Proteins with an instability index (II) below 40 are considered stable, while proteins with higher values are considered unstable (Ranjan et al., 2024). All studied chitinases had an instability index below 40, except for chitinase CHIT2 from isolate BV-ECA 31 (II 40.90), being the only chitinase classified as unstable. However, the estimated half-lives for all chitinases were greater than 20 h, according to the N-terminal rule and using the yeast model as a reference (Supplementary Table 3).

The aliphatic index (IA) of the CHIT1.1 chitinases varied between 75.43 and 85.37, while the value fluctuated between 61.60 and 61.83 for CHIT1.2 chitinases. CHIT2 chitinases exhibited IA values between 88.90 and 90.03. This suggests that all chitinases were considered thermally stable (Supplementary Table 3).

Both CHIT1 and CHIT2 chitinases showed negative values in the grand average of hydropathicity (GRAVY) parameter, indicating all chitinases are hydrophilic (Kyte and Doolittle, 1982; Uddin et al., 2020) (Supplementary Table 3).

## 4 Discussion

As a first approach toward its future exploitation, our work aimed to compare gene and protein sequences of two types of chitinases obtained from Neotropical isolates of *B. bassiana*. To determine their structural and functional characteristics and facilitate the understanding of their biological functions, we identified, manually curated and structurally annotated these sequences, as well as characterized their physical and biochemical characteristics. From the 16 chitinase genes identified in this study, we determined that chitinase 2 is more homogeneous than chitinase 1. This was evident based on the multiple sequence alignment and the dendrogram analysis of the protein sequences. We observed CHIT1.1 chitinases were similar to chitinases from isolates from South Korea (JEF-350: GenBank ID GCA_021365345.1) and Denmark (ARSEF8028: GenBank ID GCA_001682635.1); whereas CHIT1.2 chitinases grouped with isolates from South Korea (JEF-007: GenBank ID GCA_002871155.1) and China (D1-5: GenBank ID GCA_000770705.1). CHIT1.2 chitinases from Neotropical isolates grouped closer together to CHIT1.1 where isolate ECA-31 clustered with chitinases from other latitudes. This closer relationship of chitinase 1.1 from isolate ECA-31 suggests the genetic variations of *B. bassiana* may not necessarily be related to its geographic origin. In previous research, a low relationship between the geographic origin and genetic variation in Neotropical isolates of *B. bassiana* had been observed, including those analyzed in this study (Castro-Vásquez et al., 2021; Solano-González et al., 2023). It is possible to infer the presence of a potential paralog of CHIT1 (as seen in the CHIT.1 and CHIT1.2 gene groups). The presence of potential paralogous is relevant as higher number of copies with pathogenic function will impact the effectiveness of its infection processes (Gasmi et al., 2021), increasing the virulence of the fungus, therefore a relevant parameter to assess for future biological control applications. Chitinase g5795 BV-ECA is considered an outlier because, during the sequence retrieval, a frameshift was observed in the open reading frame, resulting in a protein with a start codon different from methionine. Therefore, to include this chitinase in the analysis, it was decided to define the start of the sequence at the nearest start codon, resulting in a chitinase with a significantly lower number of amino acids compared to the others.

Signal peptides are short sequences of amino acids that direct proteins toward a specific destination to fulfill a biological function (Owji et al., 2018; Teufel et al., 2022). The presence of the Secretory (Sec) Pathway and Signal Peptide (SEC/SPI peptide) in chitinases CHIT1.1 and CHIT1.2 indicated that these enzymes are likely secreted extracellularly. Although the presence of the peptide was not detected in all sequences of the chitinase 1.1 group, there is experimental evidence suggesting the involvement of chitinase CHIT1 in the infection process, indicating that this enzyme performs extracellular functions (Fang et al., 2005; Al Khoury et al., 2019). In contrast, none of the CHIT2 chitinases showed the presence of the signal peptide, suggesting that it may be an endogenous chitinase with functions related to morphogenesis and cell growth rather than being involved in the infection process. This could be associated with the high conservation of the CHIT2 sequences observed in the alignment, as being involved in basic functions for fungal development and might not require frequent environmental adaptations. We hypothesize that CHIT1 was exposed to greater evolutionary pressure due to interactions with the host and the environment (Vilcinskas, 2010), which in turn explains the variability among CHIT1 chitinases. Therefore, for the purposes of biological pest management, researchers and bioproduct developers should focus on the diverse extracellular chitinases, as these specifically interact with the host.

Functional analysis revealed that chitinases from all three groups (CHIT1.1, CHIT1.2, and CHIT2) possess the characteristic catalytic domain of glycosyl hydrolase family 18. Furthermore, they were classified within the same superfamily and subgroup of type II chitinases. Based on these findings, it is likely that CHIT1.1 and CHIT1.2 chitinases exhibit conserved functions and are products of a gene duplication event. However, the accumulation of amino acid sequence variations over time may introduce differences in physicochemical properties and secondary structures, leading to functionally similar proteins that could facilitate the adaptation of the fungus to diverse environmental conditions or different hosts.

Observed differences between the sequences of CHIT1.1 and CHIT1.2 are also reflected in their physicochemical characteristics. The latter is more water-soluble compared to the former, due to its lower GRAVY value (Kyte and Doolittle, 1982). Additionally, CHIT1.2 has a considerably lower aliphatic index, suggesting lower thermostability (Ikai, 1980). Differences were also identified in the isoelectric point (pI) and instability index; CHIT1.2 showed a less acidic pI and a lower instability index, which is associated with a longer *in vivo* half-life (Gasteiger et al., 2005). Interestingly, CHIT2 exhibited physicochemical parameters similar to CHIT1.1. The pI and GRAVY index values did not show significant differences between these two chitinases, unlike the comparisons between CHIT1.1 and CHIT1.2, or between CHIT1.2 and CHIT2. However, the latter showed the highest aliphatic index value, which can be associated with high thermal stability (Ikai, 1980). This characteristic may be of interest for biotechnological applications, as highly thermostable chitinases better withstand high temperatures and, therefore, have potential applications in fields such as agriculture, medicine, and the environment (Mathew et al., 2021). However, as it is likely an intracellular protein, its production may pose a greater challenge, as it requires genetic manipulation to direct its secretion extracellularly.

Liu et al. (2021) demonstrated that when exposed to different cuticle extracts, *B. bassiana* adjusted its infection strategy in response to new hosts. This adaptation involved differential regulation of chitinase expression within the same subgroup. In other words, the strain expressed different chitinases depending on the host being infected. Given *B. bassiana’s* broad host range (Oda et al., 2014), this plasticity may be linked to the fungus’s ability to vary its infection strategy. Moreover, this aligns with the results herein, regarding the abundance of GH18 family chitinases in this entomopathogen (Xiao et al., 2012).

Databases are invaluable resources for understanding the structural and functional characteristics of target proteins. However, the hierarchical nature of database annotations often results in non-specific or redundant information, hindering precise functional analysis, as consistently observed in our study. On the other hand, secondary structure analysis revealed a similar composition among chitinases from all three groups, suggesting significant structural conservation. Interestingly, chitinase CHIT1.2 exhibited a secondary structure composition more closely resembling CHIT2 than CHIT1.1. Previous studies by Bhagwat et al. (2021) on 15 *B. bassiana* chitinases yielded similar results, with a predominance of random coils and alpha helices. Given their shared ancestry, it is likely that the observed variations in secondary structure reflect the accumulation of changes over time, leading to functional adaptations.

There is abundant information related to the production of chitinases derived from fungi, however, reports detailing their purification and characteristics are scarce (Tupe et al., 2022). One of the study’s objectives was to provide resources to the scientific community that facilitate the development of future research in genetic and protein engineering. To serve as a foundation, we initially assessed the intrinsic physicochemical properties of chitinases knowing these parameters is significant for purification and isolation purposes (Ranjan et al., 2024), protein folding (led by hydrophobicity features) (Pinnamaneni et al., 2011; Stoykov et al., 2015), molecule interaction, functionality under variations of temperature (Islam et al., 2015) and pH conditions (Gomaa, 2021).

*B. bassiana* is a fungus known for its ability to affect a wide variety of hosts. However, different strains of this fungus show varied levels of virulence (Ortiz-Urquiza and Keyhani, 2016). This variability in virulence can pose a challenge for the commercialization of mycoinsecticides and their establishment as a strategy in integrated pest management. Gasmi et al. (2021) emphasized the importance of evaluating the genetic diversity of isolates and their effects on fungal performance. Similarly, Zhang et al. (2020) suggested that the study of genetic variants could be useful for improving the understanding of the different levels of virulence displayed by *B. bassiana* isolates towards their hosts. In the present study, we found possible genetic variants in the CHIT1 and CHIT2 chitinase sequences of Neotropical isolates. These variants could be the subject of further research to determine their possible effect on the phenotypic characteristics of the isolates, as well as the level of entomopathogenic potency among them.

## Data Availability

The original contributions presented in the study are included in the article/Supplementary Material, further inquiries can be directed to the corresponding author.

## Supplementary material

SUPPLEMENTARY FIGURE 1 Genetic structure of chit1 in ECA-UNA isolates. The green color represents the exon while the dashed lines represent the introns.

SUPPLEMENTARY FIGURE 2 Genetic structure of chit2 in ECA-UNA isolates. The green color represents the exon while the dashed lines represent the introns (absent in this case).

SUPPLEMENTARY TABLE 1 Genes recovered from the search for matches between the ECA-UNA fungal genomes and the NCBI chitinases chit1 and chit2.

SUPPLEMENTARY TABLE 2 Online blastx results for the verification of the identity of complete gene sequences.xlsx.

SUPPLEMENTARY TABLE 3 In silico physicochemical properties of chitinase identified in Neotropical isolates of B. bassiana ECA-UNA.xlsx.

SUPPLEMENTARY TABLE 4 Secondary structure analysis for CHIT1.1, CHIT1.2 and CHIT2.xlsx.
