# Supplementary figures and images for: Computational identification and characterization of chitinase 1 and chitinase 2 from neotropical isolates of Beauveria bassiana
Source: Front Bioinform. 2024 Oct 18;4:1434442. doi: 10.3389/fbinf.2024.1434442 (PMC11527780; doi:10.3389/fbinf.2024.1434442)

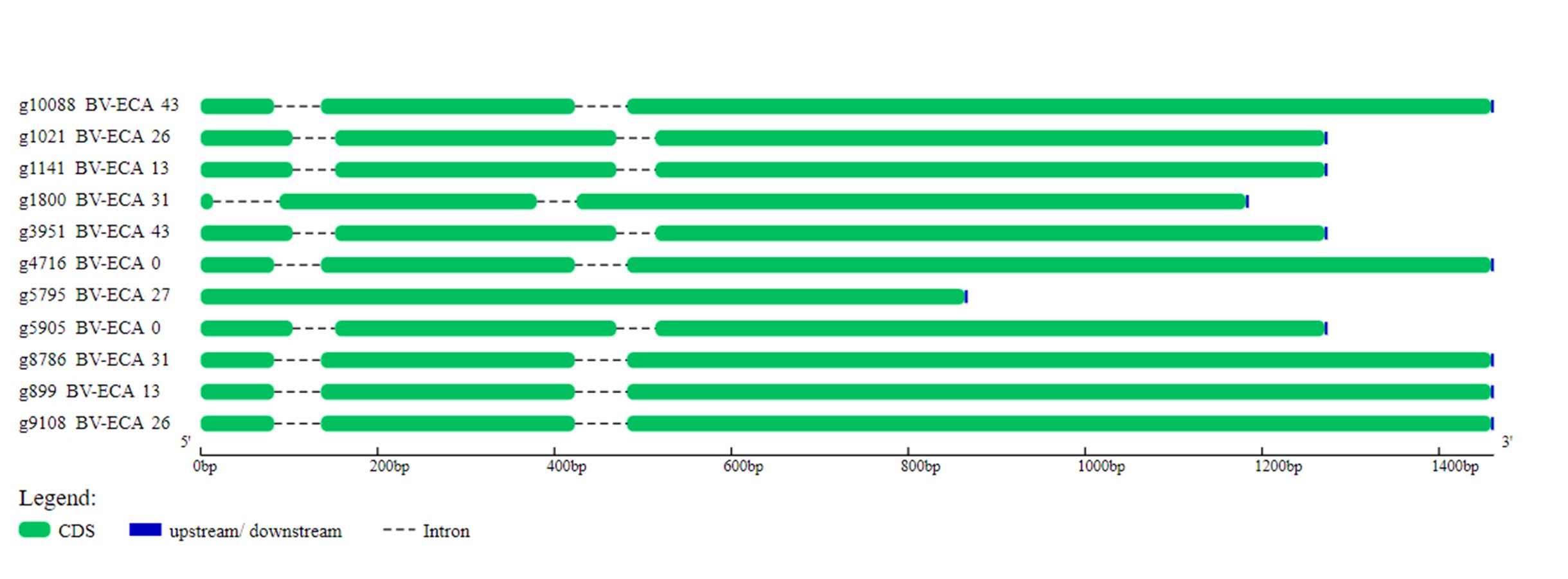

Supplement: Supplementary file 3 [file Image1.jpeg]

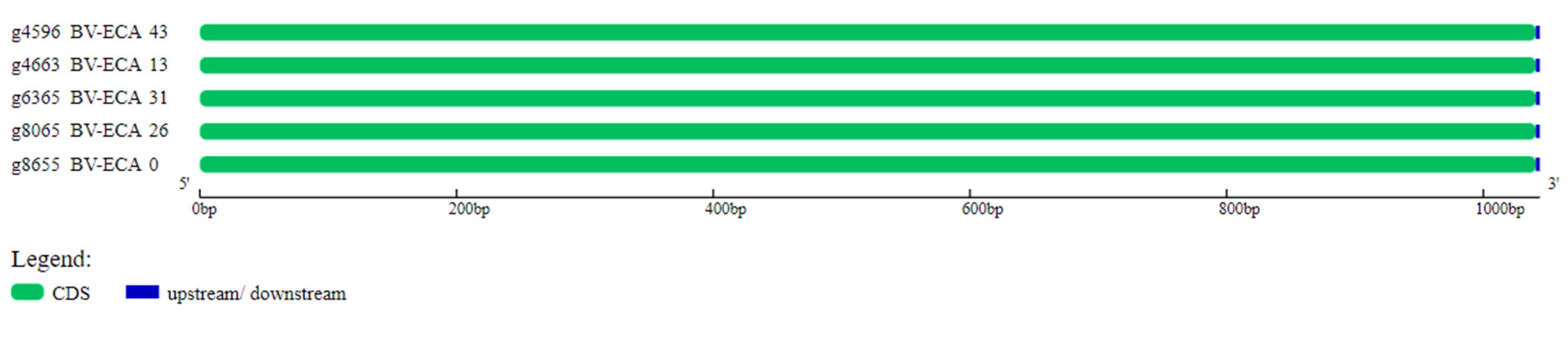

Supplement: Supplementary file 4 [file Image2.jpeg]
